# The impact of gender-affirming hormone therapy on nutrition-relevant biochemical measures

**DOI:** 10.3389/fnut.2024.1339311

**Published:** 2024-04-05

**Authors:** Jennifer Waters, Whitney Linsenmeyer

**Affiliations:** ^1^School of Health Studies, Northern Illinois University, Dekalb, IL, United States; ^2^Department of Nutrition and Dietetics, Saint Louis University, Saint Louis, MO, United States

**Keywords:** transgender, nutrition, biochemical, hormone therapy, dyslipidemia, cardiometabolic, hepatorenal, hematologic

## Abstract

Gender-affirming hormone therapy carries the potential risk for shifts in biochemical markers that may impact cardiometabolic, hematologic, hepatic, and renal health. The critical evaluation of biochemical data is an integral part of a comprehensive nutrition assessment; therefore, nutrition professionals should be aware of shifts that are expected during the course of masculinizing and feminizing hormone therapy. Changes in important biochemical values along with binary sex-specific standards for interpreting laboratory data can pose significant challenges for nutrition professionals working with transgender and gender-diverse patients who receive gender-affirming hormone therapy. Overall, research on the biochemical impact of masculinizing and feminizing hormone therapy is nascent and limited. Methodologies and outcomes measured are heterogenous across studies, introducing complexities that impede researchers from drawing definitive conclusions. In light of these limitations, this narrative review aims to describe the potential implications of masculinizing and feminizing hormone therapy regimens on biochemical measures that may influence nutrition strategies and interventions to promote optimal health.

## 1 Introduction

Nutrition professionals in the United States utilize the Nutrition Care Process (NCP) as a framework for providing nutrition care (1). The NCP is divided into four steps including nutrition assessment/reassessment, nutrition diagnosis, nutrition intervention, and nutrition monitoring and evaluation. An important domain within the nutrition assessment step of NCP is the evaluation of biochemical data, tests, and procedures. Information collected during the nutrition assessment step may influence subsequent steps within the NCP. For example, biochemical data collected as part of a nutrition assessment may be used to support a nutrition diagnosis, influence which nutrition goals or interventions are chosen, and drive decisions about what data should be monitored and evaluated when progress is later reassessed (1). The importance of nutrition in the overall care of people who are transgender and gender diverse (TGGD) has been recently underscored and presents an opportunity for nutrition professionals to expand their knowledge and skills (2–6).

TGGD individuals may choose to transition to align their appearance with their gender identity. While there are many ways someone can transition, medically transitioning is one option which involves the use of masculinizing or feminizing hormone therapy (HT) such as testosterone or a combination of estrogen and anti-androgens, respectively (7–9). Medical transition through the administration of exogenous hormones is often referred to as gender-affirming hormone therapy (GAHT). When nutrition professionals work with TGGD individuals, unique challenges may arise related to the interpretation of biochemical data as part of a comprehensive nutrition assessment. These challenges are often due to established sex-specific reference standards for interpreting certain biochemical markers (6). Without evidence-based standards to guide the nutritional care of transgender and gender-diverse individuals, it can be difficult for nutrition professionals to decide which sex-specific standards to use when analyzing biochemical data. As an additional layer of complexity, GAHT may result in changes in certain biochemical measures over time. Further, there are nutrition-relevant conditions that may concomitantly occur with GAHT including insulin resistance, diabetes, dyslipidemia, hypertension, and other biochemical alterations (7–9).

The application of an appropriate nutrition intervention is dependent on a critical analysis of nutrition-relevant biochemical values along with other assessment data such as anthropometric measurements, a detailed history of dietary intake, and pertinent medical history. Together, these vital assessment components are used to formulate interventions that promote improved health and nutrition status (1). The biochemical data selected to examine in this review are directly related to the care provided by nutrition professionals and are often readily attainable in the clinical setting. Additionally, many of the biochemical measures examined are underscored in the current iteration of the World Professional Association for Transgender Health (WPATH) Standards of Care for the Health of Transgender and Gender Diverse People, Version 8 (8). Monitoring serum lipid levels as well as measures related to blood glucose control are of significant relevance for the prevention and management of cardiometabolic conditions. Nutrition professionals can offer dietary counseling and education for reducing risk factors associated with cardiovascular disease and diabetes (10–12). Nutrition professionals are also accustomed to analyzing hematologic, hepatic, and renal biochemical data which provide insight on the development of nutritional anemias, hepatobiliary disease, and issues with hydration status/kidney function, respectively. As part of a multidisciplinary care team, it is within the scope of practice for nutrition professionals to offer strategies to help manage alterations in nutrition-relevant biochemical data (13).

The aim of this narrative review is to describe the available research published within the past 10 years that evaluates the impact of GAHT on nutrition-relevant biochemical data. A comprehensive search was conducted using PubMed, CINAHL, Cochrane, and ProQuest databases for studies published in English between January 1, 2012, and April 30, 2022. Hand-searching and reference mining were also used. Titles and abstracts were reviewed for relevance. Full text versions were reviewed as needed to conclude relevance. Studies that were irrelevant were excluded. Study designs and selected participant characteristics are summarized for studies included in this review on transgender adults in Table 1.

**Table 1:** A summary of the study design and selected participant characteristics for each study reviewed.

References	Study design (time frame)	Sample size (n)	Average age at baseline (years)	Average BMI at baseline (kg/m^2^)	HT regimen
Allen et al. (14)	Multicenter retrospective (baseline to 5 years)	91 transmen 126 transwomen	27.8 31.1	– –	Testosterone. Data on dosages and route of administration not provided. Estradiol. Data on dosages and route of administration not provided. Spironolactone. Data on dosages and route of administration not provided.
Aranda et al. (15)	Prospective observational (baseline to 12 months)	12 transmen 6 transwomen	27.1 18.8	22.5 19.6	IM testosterone undecanoate (1,000 mg/ 2–3 months) or TD testosterone (50 mg/day) Estradiol valerate (2–4 mg/day). Data on route of administration not provided. Cyproterone acetate (50 mg/day). Data on route of administration not provided.
Aranda et al. (16)	Prospective observational (baseline to 12 months)	20 transmen	27.1	22.4	IM testosterone undecanoate (1,000 mg/2–3 months) or TD testosterone (50 mg/day)
Auer et al. (17)	Prospective observational (baseline to 12 months)	6 transmen 14 transwomen	– –	23.5 23.9	Testosterone undecanoate (1,000 mg/3 months) Data on route of administration not provided. Oral estradiol valerate (4 mg/day) or TD 17-β- estradiol patch (100 μg /24 h) Oral cyproterone acetate (50 mg/day)
Auer et al. (18)	Prospective observational (baseline to 12 months)	24 transmen 45 transwomen	27.5 34.8	24.0 23.8	Testosterone undecanoate (1,000 mg/3 months). Data on route of administration not provided. Oral estradiol valerate (4 mg/day) or TD 17-β- estradiol patch (100 μg/24 h) Cyproterone acetate (50 mg/day). Data on route of administration not provided.
Broulik et al. (19)	Retrospective observational	35 transmen	48.2	25.7	Testosterone isobutyrate (1 × 25 mg/week) or Testosterone undecanoate (4×40 mg/day). Data on route of administration not provided.
Chan et al. (20)	Retrospective observational (baseline to 6 years)	34 transmen	33.0	31.0	IM/SC testosterone cypionate or IM/SC testosterone enanthate (up to125 mg/week).
Chen et al. (21)	Multicenter prospective (baseline to 3 months)	30 transmen 29 transwomen	22.0 26.0	23.2 22.1	IM testosterone esters (250 mg/2–3 weeks) or testosterone gel (50 mg/day) Oral estradiol valerate (4 mg/day) or TD estradiol patch (100 mg/24 h). Cyproterone acetate (50 mg/day). Data on route of administration not provided.
Colizzi et al. (22)	Prospective observational (baseline to 24 months)	43 transmen 79 transwomen	28.8 30.2	21.0 21.8	IM testosterone ester depot (250 mg /21.6 ± 3.17 days) TD estradiol gel (2.12 ± 0.57 mg/day) Oral cyproterone acetate (100 mg/day)
Defreyene et al. (23)	Multicenter prospective	192 transmen 239 transwomen	22.5 28.5	24.4 23.4	IM testosterone undecanoate (1,000 mg/12 weeks) or TD testosterone gel (50 mg/day) or testosterone esters (250 mg/2 weeks). Oral estradiol valerate (4 mg/day) or TD estradiol patch (50–100 μg/24 h). Oral cyproterone acetate (50 mg/day).
Deutsch et al. (24)	Prospective observational (baseline to 6 months)	31 transmen 16 transwomen	27 29	29.1 24.8	SC testosterone cypionate (50–70 mg/week) or 1% testosterone gel (5 mg/day) or TD testosterone (4 mg/day). SL micronized 17-β estradiol (4 m/day) or TD 17-β- estradiol patch (100 μg/day) or IM estradiol valerate (20 mg/ 2 weeks). Spironolactone (100–200 mg/day). Data on route of administration not provided.
Fernandez and Tannock (25)	Retrospective observational (baseline to 18 months)	19 transmen 33 transwomen	27 31	28.1 28.8	IM testosterone (average dose of 11 mg/day). Oral estrogen (average dose 1.44–1.71 mg/day). Oral spironolactone (100 mg/day).
Hashemi et al. (26)	Multicenter retrospective	438 transmen 624 transwomen	– –	– –	Data on type of HT medication, route of administration, and dosages were not provided.
Humble et al. (27)	Retrospective observational (baseline to 12 months)	150 transmen 152 transwomen	24.2 31.8	29.6 28.5	IM or SC testosterone or TD testosterone or a combination of the above regimens. Medication names and dosages not provided. Oral/SL estrogen or TD estrogen or IM estrogen or combinations of the above regimens. Medication names and dosages not provided. Oral spironolactone. Dosage not provided.
Kurahashi et al. (28)	Prospective observational	160 transmen	24.9–28.1	–	IM testosterone enanthate (125–250 mg/2–3 weeks)
Liu et al. (29)	Retrospective observational (baseline to 12 months)	65 transmen 45 transwomen	27.9 26.0	22.6 20.6	IM testosterone cypionate (average dose 166.6 mg/2 weeks) Conjugated estrogen. Dosage and route of administration not provided. Cyproterone acetate once daily if indicated. Dosage and route of administration not provided.
Maheshwari et al. (30)	Retrospective observational (baseline to 12 months)	24 transmen 84 transwomen	23.0 30.0	37.2 26.6	IM/SC testosterone (50–60 mg/week). Name of medication and dosage not provided. Oral/SL estradiol (4–6 mg/day) or injectable estradiol (2–5.62 mg/weekly) or TD estradiol (0.1–0.3 mg/day)
Manieri et al. (31)	Prospective observational (baseline to 12 months)	27 transmen 56 transwomen	30.2 32.7	– –	ITD testosterone gel (25 mg/day) or IM testosterone enanthate (100 mg/10–15 days) or IM testosterone undecanoate (1,000 mg/3–4 months) Oral or TD 17-β- estradiol (2–6 mg/day). Cyproterone acetate (50–100 mg/day) or spironolactone (100–200 mg/day) or dutasteride (0.5 mg/day). Administration route not provided.
Martinez et al. (32)	Retrospective observational (baseline to 24 months)	21 transmen 19 transwomen	23.9 23.9	24.0 23.8	Data on type of HT medication, route of administration, and dosages were not provided.
Quirós et al. (33)	Retrospective observational (baseline to 24 months)	97 transmen 150 transwomen	28.6 32.4	24.0 24.1	TD testosterone gel (50 mg/5 g per day) or IM testosterone undecanoate (1,000 mg/month) Oral conjugated equine estrogen or oral estradiol valerate or TD estradiol. Dosages not provided. Cyproterone or flutamide (individualized dosages) Data on dosages or routes of administration not provided.
Shadid et al. (34)	Multicenter prospective (baseline to 12 months)	35 transmen 55 transwomen	26.1 34.4	23.2 23.7	IM testosterone undecanoate (1,000 mg/12 weeks). Oral estradiol valerate (4 mg/day) or TD estradiol patch (100 μg/72 h) or TD estrogen gel (3 mg/day). Oral cyproterone acetate (50 mg/day)
SoRelle et al. (35)	Multicenter retrospective (baseline to 6 months)	62 transmen 87 transwomen	27.0 31.0	28.0 27.0	Testosterone cypionate (35–300 mg) or TD testosterone or combinations of the above regimens. Frequency of dose not provided. Oral estradiol (2–8 mg) or estradiol valerate (5–40 mg) or conjugated estrogens (2.5 mg) or estradiol cypionate (2–5 mg) or TD estradiol (0.5 mg) or combinations of the above regimens. Frequency of dose not provided. Spironolactone (25–300 mg) or cyproterone (50 mg). Frequency of dose and route of administration not provided.
van Velzen et al. (36)	Prospective observational (baseline to 12 months)	188 transmen 242 transwomen	26.4 32.3	23.5 22.8	IM/SC testosterone undecanoate (1,000 mg/12 weeks) or TD testosterone gel (50 mg/day) or IM/SC testosterone esters (250 mg/2 weeks). Oral estradiol valerate (4 mg/day) or TD estradiol patch (100 μg/24 h). Cyproterone acetate (50 mg/day). Route of administration not provided.
Vita et al. (37)	Retrospective observational (baseline to 30 months)	11 transmen 21 transwomen	25.1 25.2	22.6 22.5	IM testosterone enanthate or IM testosterone undecanoate. Dosages not provided. Oral estradiol valerate (2–6 mg/day) Cyproterone acetate (50–100 mg/day). Route of administration not provided.
Wierckx et al. (38)	Multicenter prospective (baseline to 12 months)	53 transmen 53 transwomen	21.7–27.3 19.3–31.7	24.8 23.1 (oral) 26.1 (TD)	IM testosterone undecanoate (1,000 mg/12 weeks) or if not tolerated, IM testosterone esters. Oral estradiol valerate (4 mg/day) or TD 17-β estradiol patch (100 μg/24 h) or TD 17-β estradiol gel (4 mg/day) Cyproterone acetate (50 mg/day). Route of administration not provided.

BMI, body mass index; HT, hormone therapy; IM, intramuscular; TD, transdermal; SC, subcutaneous; SL, sublingual.

## 2 Impact on of GAHT cardiometabolic biochemical markers

Individuals pursuing GAHT may develop cardiometabolic risk factors during the course of therapy (8). Comorbidities that may emerge include hypertension (39, 40), cardiovascular disease (40–44), and diabetes (39–47). These risk factors develop commonly during both masculinizing and feminizing hormone therapy regimens (8). Additionally, certain cardiometabolic events are a potential risk for those on GAHT regimens when compared to individuals not pursuing medical transition. These events may include myocardial infarction (39–41, 44, 48), stroke (40, 41), and venous thromboembolism (49). While these cardiometabolic events appear to be more prominent in individuals undergoing feminizing HT, they can also manifest for individuals on masculinizing HT regimens (8). The potential risk for these conditions and events are thought to be related in part to alterations in serum lipid levels that onset with the initiation of hormone therapy (50–53) as well as unfavorable effects of GAHT on blood pressure and glycemic control (18, 34, 50, 53, 54). Anthropometric changes resulting from GAHT may also influence the development of cardiometabolic biochemical alterations and risk factors. These include an increase in body mass index (BMI) (15, 16, 18, 22, 24, 25, 29, 32–34) and waist circumference (22, 34) in individuals pursuing masculinizing HT, and an increase in BMI (22, 32–34) and body fat composition (17, 18, 34, 38, 55–57) for those on feminizing hormone regimens. An understanding of the cardiometabolic risks associated with GAHT can prepare nutrition professionals to provide early and preventative nutrition counseling to clients who are interested in initiating GAHT to support medical transition.

### 2.1 Total cholesterol

There are mixed results within the current literature regarding changes in serum total cholesterol (TC) while receiving GAHT (Table 2). In several studies where serum TC was compared at baseline with subsequent measurements at various time intervals after initiation of hormones in adult subjects, there were no significant differences observed for those receiving masculinizing (14, 17, 18, 24, 25, 27, 31, 34, 35, 37) or feminizing hormone regimens (14, 17, 24, 25, 27, 29, 31, 33, 35, 37). While most researchers have not observed a difference in TC in adult subjects undergoing GAHT, a few studies exist in which significant changes were reported (16, 18, 22, 29, 33, 34, 38).

**Table 2:** A summary of the changes in serum lipid levels in adults receiving masculinizing or feminizing hormone therapy.

References	Number of participants	Changes in biochemical values							
		Masculinizing HT				Feminizing HT			
		TC	LDL-C	HDL-C	TRIG	TC	LDL-C	HDL-C	TRIG
Allen et al. (14)	91 transmen; 126 transwomen	NC	↑	↓	NC	NC	NC	↑	NC
Aranda et al. (15)	12 transmen; 6 transwomen	NC	↑	↓	–	NC	NC	↑	–
Aranda et al. (16)	20 transmen	↑	↑	↓	↑	–	–	–	–
Auer et al. (17)	6 transmen; 14 transwomen	NC	NC	NC	NC	NC	NC	↑	NC
Auer et al. (18)	24 transmen; 45 transwomen	NC	↑	↓	NC	↓	↓	NC	↓
Chan et al. (20)	34 transmen	–	NC	NC	NC	–	–	–	–
Colizzi et al.(22)	43 transmen; 79 transwomen	↑	↑	↑	↑	↑	↑	↓	↑
Deutsch et al. (24)	31 transmen; 16 transwomen	NC	NC	↓	NC	NC	NC	↑	↑
Fernandez and Tannock (25)	19 transmen; 33 transwomen	NC	NC	NC	NC	NC	NC	↑	NC
Humble et al.(27)	150 transmen; 152 transwomen	NC	NC	↓	NC	NC	NC	↑	NC
Liu et al. (29)	65 transmen; 45 transwomen	↓	↑	↓	NC	NC	NC	NC	NC
Manieri et al. (31)	27 transmen; 56 transwomen	NC	NC	NC	NC	NC	NC	NC	NC
Martinez et al. (2018) (32)	21 transmen; 19 transwomen	–	–	–	NC	–	–	–	↑
Quirós et al. (2015) (33)	97 transmen; 150 transwomen	↑	↑	↓	↑	NC	NC	NC	NC
Shadid et al. (34)	35 transmen; 55 transwomen	NC	↑	↓	NC	↓	↓	↓	↓
SoRelle et al. (35)	62 transmen; 87 transwomen	NC	NC	↓	↑	NC	NC	NC	NC
van Velzen et al. (36)	188 transmen; 242 transwomen	–	↑	↓	↑	–	↓	↓	↓
Vita et al. (37)	11 transmen; 21 transwomen	NC	NC	NC	NC	NC	NC	NC	NC
Wierckx et al. (38)	53 transmen; 53 transwomen	↑	↑	↓	↑	↓	↓	↓	↓

HT, hormone therapy; TC, total cholesterol; LDL-C, low-density lipoprotein cholesterol; HDL-C, high-density lipoprotein cholesterol; TRIG, triglycerides; NC, no significant change; ↑, significant increase; ↓, significant reduction.

For adults receiving masculinizing HT, there is evidence that an increase in TC may be observed (16, 22, 33, 38). This biochemical shift may manifest as early as 6 months after HT initiation (16), however has been more commonly observed at 12- or 24-months post-initiation (16, 22, 33, 38). Participants were typically prescribed injectable testosterone esters, most commonly intramuscular testosterone undecanoate, with some participants using either injectable or transdermal preparations of testosterone (Table 1). In contrast to previous evidence, recent research conducted by Liu et al. (29) reported a reduction in mean serum TC that occurred after 3–6 months following initiation of intramuscular testosterone cypionate in 65 hormone-naïve transgender men (183.4 vs. 178.2 mg/dL; *p* = 0.032). A recent large-scale study (58) comparing adherence to GAHT regimens in transgender men indicated that those who were prescribed testosterone undecanoate had a two-fold higher compliance and thus displayed more stability with their testosterone levels than those on testosterone cypionate. This may be due to the shorter-acting nature of testosterone cypionate, requiring more frequent administration. This may have been a plausible explanation for the conflicting findings, however, in Liu et al. (29) study, the participants’ testosterone levels were well above the target range of 400–700 ng/dL (8) set by WPATH standards and were at a mean of 890 ± 90 ng/dL at the 3–6-month mark. It is difficult to draw solid conclusions regarding the impact of masculinizing HT on TC levels as there are several studies in which no significant changes were observed. If a change is anticipated to occur, it may be a potential increase in TC, though more research studies with homogeneity of samples are needed to formulate stronger interpretations of the impact of masculinizing HT on TC levels.

Feminizing HT regimens are thought to have a more favorable impact on cardiovascular biomarkers such as TC, although available evidence is not consistent. When a reduction in TC has been reported in individuals pursuing feminizing HT, it generally emerges around 12 months after HT initiation (18, 34, 38). In contrast to findings that support a favorable change to TC with feminizing HT, a study of 79 transgender women reported a significant increase in mean serum TC at 12 months (161.23 vs. 181.70 mg/dL; *p* < 0.001) and 24 months (161.23 vs. 194.44 mg/dL; *p* < 0.001) after initiation of HT (22). It should be noted that in this study (22), the HT regimen included a combination of transdermal estradiol gel (2.12 ± 0.57 mg/day) and oral cyproterone acetate (100 mg/day) (Table 1). Among research in which a favorable impact on TC was observed (18, 34, 38), a lower dosage of the anti-androgen cyproterone acetate was applied (50 mg/day) and the estrogen regimen typically included oral estradiol valerate (4 mg/day) or a transdermal estrogen preparation for participants over the age of 45 years in the form of a patch (100 μg) (18, 34, 38) or a gel (3–4 mg/day) (Table 1) (34, 38). The variance in hormone regimens and dosages employed across the available research may explain the conflicting results. Based on the current available evidence, the impact of feminizing HT on TC levels is somewhat inconclusive. While available research on the impact of GAHT on cardiometabolic biochemical markers in adolescents is scarce, a few studies are focused more exclusively on this age group. There was a wide range of ages represented across these studies including participants as young as 13 years old and as old as 25 years old. Adolescents and young adults were also more commonly reported to have no change in their serum TC while receiving masculinizing (59–62) or feminizing (59, 61, 63) hormone therapy regimens. Klaver and colleagues (63) did observe a slight increase in serum TC in young adults on masculinizing hormone regimens. This study (63) was the only one included in which 100% of the participants had been treated with gonadotropin-releasing hormone agonists (also known as puberty blockers) prior to initiating testosterone, which complicates comparisons among studies and may be an explanation for the impact on TC. More research is required to better evaluate the implications of GAHT on TC in this population.

### 2.2 Low-density lipoprotein cholesterol

The impact of GAHT on serum low-density lipoprotein cholesterol (LDL-C) has been studied extensively in adults with mixed results (Table 2). In many studies, no significant changes in LDL-C were observed for transgender adults receiving masculinizing (17, 20, 24, 25, 27, 31, 35, 37) or feminizing (14, 15, 17, 24, 25, 27, 29, 31, 33, 35, 37) hormone therapy regimens, while other studies have detected significant shifts in LDL-C (14, 16, 18, 22, 29, 33, 34, 36, 38).

When changes in LDL-C have been reported with masculinizing HT, it has typically involved an unfavorable increase (14–16, 18, 22, 29, 33, 34, 36, 38). These alterations may occur as early as 6 months after initiation of therapy (15, 16), however in most studies where an unfavorable change was observed, it occurred at 12 months or later (14, 15, 18, 22, 29, 33, 34, 36, 38). Intramuscular testosterone undecanoate (1,000 mg) or a form of transdermal testosterone (50 mg) were offered to participants as the masculinizing HT regimen in most of the included studies (Table 1) (15–18, 31, 33, 34, 36, 38). In many of the studies in which no change was observed in LDL-C with masculinizing HT, the type of testosterone, route of administration, and/or dosages were either not provided or not clear (25, 27, 37) or a different HT regimen was offered (20, 24, 35) (Table 1). The variability in HT regimens may offer one explanation for the conflicting findings. Current evidence suggests a potential increase in LDL-C may be anticipated for adults receiving masculinizing HT.

In studies of adults undergoing feminizing HT in which a change in LDL-C was observed, most reported a reduction over 12 months of therapy (18, 34, 36, 38). In these studies, cyproterone acetate (50 mg/day) was the anti-androgen prescribed along with either oral estradiol valerate (4 mg/day) or a transdermal estrogen in the form of a patch (100 μg) (18, 34, 36, 38) or a gel (3–4 mg/day) (Table 1) (34, 38). Using a higher dose of cyproterone acetate (100 mg/day) with a transdermal estradiol gel (2.12 ± 0.57 mg/day), Colizzi et al. (22) identified an unfavorable shift in LDL-C after 12 months (100.09 vs. 120.58 mg/dL; *p* < 0.001) and 24 months (100.09 vs. 130.05 mg/dL; *p* < 0.001). The difference in HT regimens may be the reason for the variance in findings. Based on the current available evidence, a potential for reduction in LDL-C may be anticipated with feminizing HT.

Apart from one study in which an increase after 5 years was noted (63), LDL-C levels seem to remain stable in adolescents and young adults receiving GAHT (59, 60, 62, 64). Again, the few studies that included adolescents had a wide age range and in only one study (63), all the participants had received gonadotropin-releasing hormone agonists. Findings should be interpreted with caution.

### 2.3 High-density lipoprotein cholesterol

Existing data provide sound evidence for high-density lipoprotein cholesterol (HDL-C) alterations that co-occur with masculinizing HT in adults (Table 2). With a masculinizing HT regimen, an unfavorable reduction in HDL-C may be anticipated (14–16, 18, 24, 27, 29, 34–36, 38). This effect on HDL-C may be observed as early as 3–6 months after HT initiation (14, 24, 27, 29, 35), and is often detected at 12 months post-initiation or later (14–16, 18, 27, 29, 33, 34, 36, 38). Studies in which no impact on HDL-C with masculinizing HT was observed had smaller sample sizes (*n* ≤ 35) which may explain the lack of significance (17, 20, 25, 31, 37). For adults receiving feminizing HT, it may be anticipated that a favorable rise in HDL-C will result (14, 15, 18, 24, 25, 27), although a few studies exist in which the opposite effect on HDL-C was observed (22, 34, 36, 38). Changes in HDL-C for those on feminizing HT seem to onset earlier at 3–6 months (14, 15, 24, 25, 27), however can also first emerge at 12 or more months post-initiation of HT (15, 17, 22, 27, 34, 36, 38). It is not clear why there exists such a conflict in findings among studies on individuals undergoing feminizing HT. Future research may explore the causes of variance among individuals. In adolescents and young adults, HDL-C may also drop with a masculinizing HT regimen (59, 60, 62–64) and rise with feminizing HT (61, 64).

### 2.4 Triglycerides

According to the WPATH-2022 Standards of Care (8), serum triglycerides may shift in adults on GAHT (Table 2). For those on a masculinizing hormone regimen, triglyceride levels seem to either increase (16, 22, 33, 35, 36, 38) or remain unchanged (14, 17, 18, 20, 24, 25, 27, 29, 31, 32, 34, 37). A wide variety of HT regimens were used in both studies where triglyceride levels increased as well as those in which a change was not observed (Table 1). The majority of studies (75%) reviewed in which a change in triglyceride levels was not exhibited with masculinizing HT had smaller sample sizes (n ≤ 35) (17, 18, 20, 24, 25, 31, 32, 34, 37). This may serve as one explanation for the lack of significant findings. When there is a change in serum triglycerides, it typically manifests at 12 or more months post-initiation of HT (16, 22, 33, 36, 38). While most research supports no change in serum triglycerides for adults on feminizing HT (14, 17, 25, 27, 29, 31, 33, 35, 37), studies that detected a shift had more variable results (18, 22, 24, 32, 34, 36, 38). Specific feminizing HT regimens administered were diverse across the available research. In all four studies in which a reduction in triglycerides with feminizing HT was observed, the regimen included either oral estradiol valerate (4 mg/day) or a transdermal preparation (doses and routes of administration varied) as well as cyproterone acetate (50 mg/day) (18, 34, 36, 38). Although Auer et al. (17) and Vita et al. (37) also applied similar medication regimens in their studies, there were a small number of participants (*n* = 14 and *n* = 21, respectively) which may have impeded reaching statistical significance of the findings. In adolescents and young adults, serum triglycerides may rise with either masculinizing or feminizing hormone regimens (61, 63) although there is limited evidence available and much heterogeneity among the studies. While the current evidence may support anticipating a potential increase in triglycerides with masculinizing HT in adults, the impact of feminizing HT regimens on triglyceride levels requires further exploration.

### 2.5 Glucose

Fasting glucose (FG) levels do not seem to be significantly impacted by GAHT for adults (27, 29, 31, 34, 38) (Table 3) or adolescents/young adults (61, 63). The studies included a wide range of feminizing hormone regimens. In two studies, a statistically significant increase in FG was detected for adults receiving feminizing HT (22, 35) however the results did not achieve clinical significance. There were no overt commonalities among the two studies that would differentiate them from others that lack statistically significant findings. Further exploration is needed to determine whether fasting blood glucose is impacted significantly by GAHT.

**Table 3:** A summary of the changes in fasting glucose, hemoglobin A1C, and fasting insulin levels in adults receiving masculinizing or feminizing hormone therapy.

References	Number of participants	Changes in biochemical values					
		Masculinizing HT			Feminizing HT		
		FG	A1C	FI	FG	A1C	FI
Chan et al. (20)	34 transmen	–	NC	–	–	–	–
Colizzi et al. (22)	43 transmen; 79 transwomen	–	–	↑	↑	–	↑
Humble et al. (27)	150 transmen; 152 transwomen	NC	↓	–	NC	↓	–
Liu et al. (29)	65 transmen; 45 transwomen	NC	NC	↓	NC	↑	NC
Manieri et al. (31)	27 transmen; 56 transwomen	NC	–	–	NC	–	–
Shadid et al. (34)	35 transmen; 55 transwomen	NC	–	NC	NC	–	↑
SoRelle et al. (35)	62 transmen; 87 transwomen	NC	–	–	↑	–	–
Wierckx et al. (38)	53 transmen; 53 transwomen	NC	–	↓	NC	–	↑

HT, hormone therapy; FG, fasting glucose; A1C, hemoglobin A1C; FI, fasting insulin; NC, no significant change; ↑, significant increase; ↓, significant reduction.

### 2.6 Hemoglobin A1C

Hemoglobin A1C (A1C) (Table 3) has not been extensively researched in individuals pursuing GAHT, therefore drawing solid conclusions is not feasible. Humble and colleagues (27) reported a reduction in A1C levels after 12 months of GAHT which was reflected for both masculinizing and feminizing hormone regimens. Conversely, another study indicated an increase in A1C in subjects after receiving 3–6 months of feminizing hormones (29). Interestingly, participants were younger (mean age: 26 years vs. 31.8 years) and had a lower BMI (mean: 20.6 kg/m^2^ vs. 28.5 kg/m^2^) at baseline in the study (29) where an increase in A1C was observed compared to those who had a reduction in A1C (27). Further, a different HT regimen was prescribed in each of the studies, one (27) using estrogen (oral/sublingual, transdermal, or intramuscular) with spironolactone and the other (29) conjugated estrogen with cyproterone acetate. There are multiple variables that differ making it difficult to determine any solid conclusions. Data from studies on adolescents and young adults are limited and have yet to show a significant impact of GAHT on A1C levels (59). The scarcity of homogenous research presents significant challenges in understanding the impact of GAHT on A1C levels.

### 2.7 Insulin

Fasting insulin (FI) levels may be impacted by GAHT (Table 3), however more research is necessary to strengthen the existing evidence. While Colizzi et al. (22) detected a significant increase in FI levels in adults after 24 months of masculinizing HT, other studies have reported either no change (34) or a reduction in FI at 12 (38) or 24 months (29) post-initiation of HT. For adults on feminizing HT regimens, it appears that FI may increase at 12 months (22, 34, 38) post-initiation of HT or remain unchanged (29). There are limited data available on the impact of GAHT on FI for adolescents and young adults, however Klaver and colleagues (63) reported a significant reduction in FI for young adults after receiving 5 years of masculinizing HT and no significant changes for those on a feminizing HT regimen. Overall, the etiology for the wide variability among findings is not clear. Further research with less heterogeneity would be necessary to sufficiently explore the impact of GAHT on FI levels.

### 2.8 Conclusion

The variety of hormone preparations and dosages used among participants in studies where changes in cardiometabolic biochemical markers were examined pose challenges in making clear inferences. Further, some studies only included participants who were hormone-naïve, meaning they had never received GAHT prior to the study’s initiation (16, 18, 29, 34, 38), while others either included some participants who had received GAHT previously (17, 20, 33) or it was not clear whether researchers excluded participants for prior exposure to GAHT (22, 27, 31). Exclusion criteria in general varied widely across studies. In much of the research reviewed, potential subjects were excluded from participation for a history of cardiovascular disease or associated risk factors (16, 22), dyslipidemias (18, 22, 29), diabetes or hyperglycemia (16, 18, 22), however in some studies the participants were not excluded from analyses for these potentially confounding factors (33, 38). Additionally, BMI and age at baseline, which are variables that could impact cardiometabolic biochemical values, varied across studies. Despite the lack of homogeneity and the limitations within the current body of literature, some trends in the existing data may help healthcare professionals anticipate changes in cardiometabolic biomarkers. In adults undergoing masculinizing HT, the potential exists to observe an unfavorable increase in TC, LDL-C, and triglycerides; and a reduction in HDL-C. In adults on feminizing regimens, a favorable reduction in LDL-C may be anticipated. There is not enough congruent evidence at this time to support anticipating changes in FG, A1C, or FI in adults on GAHT or in TC, HDL-C, or TG with feminizing regimens.

## 3 Impact of GAHT on hematologic biochemical markers

Testosterone has an erythropoietic effect and thus it is expected to observe an increase in biochemical values like hemoglobin and hematocrit when one is on a masculinizing hormone regimen (9). For individuals pursuing masculinizing HT, it is recommended that hemoglobin and hematocrit are assessed at baseline and every 3 months after initiation of HT for the first year and then at least annually thereafter (7, 9). When evaluating hemoglobin and hematocrit levels for patients on GAHT, it is important to use appropriate reference ranges. For individuals on masculinizing hormone regimens and who are amenorrheic, it is recommended that the male reference value is used for the lower limit of the normal reference ranges. The male reference value is recommended for the upper limit of the normal ranges (9). For transgender women, it is important to consider the reduction in testosterone as well as lack of menses. Additionally, if gonads are retained, there may be pulsatile testosterone activity. When assessing hemoglobin and hematocrit levels for those on feminizing HT, the female reference value should be used for the lower limit of the normal reference range and the male reference value for the upper limit (9).

### 3.1 Hemoglobin

Robust evidence exists to support that masculinizing HT regimens may result in an increase in hemoglobin (25, 27–29, 33, 35, 37) and a reduction for those receiving feminizing hormones (27, 29, 33, 35, 37) (Table 4). These shifts in hemoglobin may be detected as early as 3–6 months after HT initiation (25, 27–29, 35). In adolescents and young adults, the same impact on hemoglobin levels has been observed for masculinizing (59, 61, 65) and feminizing HT regimens (61, 65).

**Table 4:** A summary of the changes in hematologic biochemical data in adults receiving masculinizing or feminizing hormone therapy.

References	Number of participants	Changes in biochemical values							
		Masculinizing HT				Feminizing HT			
		Hgb	Hct	MCV	RBC	Hgb	Hct	MCV	RBC
Chan et al. (20)	34 transmen	–	↑	–	–	–	–	–	–
Defreyene et al. (23)	192 transmen; 340 transwomen	–	↑	–	–	–	↓	–	–
Fernandez and Tannock (25)	19 transmen; 33 transwomen	↑	↑	–	↑	–	–	–	–
Humble et al.(27)	150 transmen; 152 transwomen	↑	↑	NC	↑	↓	↓	NC	↓
Kurahashi et al. (28)	160 transmen	↑	↑	–	↑	–	–	–	–
Liu et al. (29)	65 transmen; 45 transwomen	↑	↑	–	–	↓	↓	–	–
Manieri et al. (31)	27 transmen; 56 transwomen	NC	NC	–	–	NC	NC	–	–
Quirós et al. (33)	97 transmen; 150 transwomen	↑	–	–	–	↓	–	–	–
SoRelle et al. (35)	62 transmen; 87 transwomen	↑	↑	–	↑	↓	↓	–	↓
Vita et al. (37)	11 transmen; 21 transwomen	↑	↑	NC	↑	↓	↓	NC	↓
Wierckx et al. (38)	53 transmen; 53 transwomen	–	↑	–	–	–	↓	–	–

HT, hormone therapy; Hgb, hemoglobin; Hct, hematocrit; MCV, mean corpuscular volume; RBC, red blood cell count; NC, no significant change; ↑, significant increase; ↓, significant reduction.

### 3.2 Hematocrit

GAHT’s impact on hematocrit has been well-documented (Table 4). Much like hemoglobin, a rise in hematocrit is expected with masculinizing HT (20, 23, 25, 27–29, 35, 37, 38) and a reduction with feminizing HT (23, 27, 29, 35, 37, 38). Shifts in hematocrit may also be detected as early as 3–6 months after beginning GAHT (23, 25, 27–29, 35). While limited data on adolescents and young adults exist, the available evidence supports that an increase in hematocrit may be observed with masculinizing HT (59, 60).

### 3.3 Mean corpuscular volume

Limited research exists on the impact of GAHT on mean corpuscular volume (MCV). In two recent studies, there were no significant changes in MCV for adults receiving masculinizing or feminizing HT (27, 37). The availability of evidence is not sufficient to draw conclusions on the impact of GAHT on MCV levels.

### 3.4 Red blood cell count

As expected with a rise in circulating testosterone, red blood cell (RBC) count seems to increase for adults on masculinizing HT (25, 27, 28, 35, 37) and a reduction is typically observed in adults on feminizing HT (27, 35, 37). These shifts in RBC count may be observed at 3–6 months post HT initiation (25, 27, 28, 35). In one study on adolescents receiving GAHT, an increase in RBC count was reported for both subjects on masculinizing and feminizing hormone regimens after 4 months (65). Additional research is requisite to more adequately appraise the impact of GAHT on RBC count on adolescents.

### 3.5 Conclusion

Based on the research reviewed, a rise in hemoglobin and hematocrit may be anticipated for individuals on masculinizing HT regimens, while a reduction in both values may be expected for those on feminizing HT. In only one study (31) reviewed, no changes were observed in hemoglobin or hematocrit, however the smaller sample size (*n* = 27) may have been a barrier to achieving statistical significance. Further investigation on the effects of GAHT on MCV is warranted in adults and adolescents, given the dearth of available research. Like hemoglobin and hematocrit, a rise in RBC count may be expected with masculinizing HT and a reduction with feminizing regimens.

## 4 Impact of GAHT on hepatic biochemical markers

The use of exogenous testosterone carries a moderate risk for severe liver dysfunction due to expected elevations in liver transaminases, therefore close monitoring of hepatic biochemical markers is indicated (7). It is important to use appropriate reference ranges when evaluating alkaline phosphatase levels due to potential retention or changes in lean body mass which can affect this biochemical marker. The male reference value should be used for the upper limit of the normal reference range for both masculinizing and feminizing hormone regimens (9).

### 4.1 Alkaline phosphatase

Alkaline phosphatase levels have the potential of shifting during the course of GAHT (Table 5). In one study of 11 adults receiving masculinizing HT, a significant increase in alkaline phosphatase (AP) was observed after a mean of 30 months (51.8 vs. 83.4 U/L; *p* < 0.0001) (37), whereas other research supports that AP may not significantly shift with masculinizing hormone regimens (19, 27, 35). The reason for the discrepancy in findings among the studies is ambiguous. In adults on feminizing HT, research from two studies provide evidence that a significant reduction in AP may observed after 6 months (27, 35). Humble and colleagues (27) reported an increase in AP (69 vs. 62 U/L; *p* < 0.05) in adults on feminizing HT. SoRelle et al. (35) also observed a reduction in AP with feminizing HT (73.2 vs. 62.3 U/L; *p* < 0.001). In one study (37), there was no observable shift in AP with feminizing HT. This may be attributed to a small sample size (*n* = 21). Overall, the impact of GAHT on AP has been underexplored and warrants further examination to establish consistent findings.

**Table 5:** A summary of the changes in hepatic biochemical data in adults receiving masculinizing or feminizing hormone therapy.

References	Number of participants	Changes in biochemical values									
		Masculinizing HT					Feminizing HT				
		AP	AST	ALT	TBili	Alb	AP	AST	ALT	TBili	Alb
Broulik et al. (19)	35 transmen	NC	–	–	–	–	–	–	–	–	–
Chen et al. (21)	30 transmen; 29 transwomen	–	NC	NC	–	NC	–	↓	NC	–	↓
Fernandez and Tannock (25)	19 transmen; 33 transwomen	–	NC	NC	–	–	–	NC	NC	–	–
Hashemi et al. (26)	438 transmen; 624 transwomen	–	NC	NC	–	–	–	NC	NC	–	–
Humble et al.(27)	150 transmen; 152 transwomen	NC	NC	↑	–	–	↓	NC	NC	–	–
Kurahashi et al. (28)	160 transmen	–	NC	NC	–	–	–	–	–	–	–
Liu et al. (29)	65 transmen; 45 transwomen	–	–	NC	–	–	–	–	NC	–	–
SoRelle et al. (35)	62 transmen; 87 transwomen	NC	↑	↑	NC	NC	↓	NC	↓	↓	↓
Vita et al. (37)	11 transmen; 21 transwomen	↑	NC	NC	↑	–	NC	NC	NC	NC	–
Wierckx et al. (38)	53 transmen; 53 transwomen	–	↑	↑	–	–	–	↓	↓	–	–

HT, hormone therapy; AP, alkaline phosphatase; AST, aspartate transaminase; ALT, alanine transaminase; TBili, total bilirubin; Alb, albumin; NC, no significant change; ↑, significant increase; ↓, significant reduction.

### 4.2 Aspartate transaminase

Most research exploring the impact of GAHT on aspartate transaminase (AST) has shown no significant changes for either masculinizing (21, 25–27, 37) or feminizing hormone regimens (25–27, 37) (Table 5). However, in a few studies, a shift in AST levels has been reported (21, 35, 38). In a study of adults receiving 6 months of masculinizing HT an increase in AST was detected (18 vs. 23 U/L; *p* < 0.005) (35) which aligns with a different study with similar results (20 vs. 24 U/L; *p* = 0.01) (38). The basis for inconsistencies in results across studies investigating the influence of masculinizing HT on AST is unclear. Chen and colleagues (21) observed a slight reduction in AST in adults on 3 months of feminizing HT (20 vs. 18 U/L; *p* < 0.01). Further, after 12 months of feminizing HT, Wierckx et al. (38) reported a reduction in AST (24.1 vs. 17.7 U/L; *p* < 0.001). In both studies (21, 38), cyproterone acetate was prescribed as the anti-androgen while in two (25, 27) of the four studies where no changes in AST were detected, spironolactone was the anti-androgen prescribed. In one study (26), authors did not report the medication used and in the other cyproterone acetate was provided (37). Future research endeavors may benefit from exploring the influence of various anti-androgen regimens on AST levels. In studies evaluating changes in AST in adolescents and young adults, most have reported no significant changes (59, 60, 66), however in one study, a reduction in AST was observed with masculinizing and feminizing hormone regimens (61). Overall, the limited data that exist related to the impact of GAHT on AST are not consistent nor sufficient to derive any solid insights.

### 4.3 Alanine transaminase

Much of the published research investigating alanine transaminase (ALT) levels in adults on GAHT has shown no significant impact from either masculinizing (21, 25, 26, 28, 29, 37) or feminizing HT (21, 25–27, 29, 37) (Table 5). In adults on masculinizing hormone regimens, results from three research studies support that ALT may rise after six (27, 35) or 12 months (38). Humble and colleagues (27) observed an increase from 15.9 to 18.7 U/L (*p* < 0.01) over six months, whereas SoRelle et al. (35) reported a more clinically significant increase from 17 to 23 U/L (*p* < 0.001) with six months of masculinizing HT. After 12 months of masculinizing HT, another study exhibited an increase in ALT from 16 to 20 U/L (*p* = 0.02) (38). For adults receiving feminizing HT, a reduction in ALT was observed in two different studies at six months (35) (21 vs. 18 U/L; *p* < 0.005) and 12 months (38) (25.0 vs. 18.6 U/L; *p* = 0.01). Most of the research on ALT in adolescents and young adults on GAHT has indicated that shifts are not expected for masculinizing (59, 60, 66) or feminizing regimens (61, 66). However, minimal data support that a reduction of ALT may be observed with feminizing HT (59) and an increase with masculinizing HT (61). The rationale for the variations in findings among studies examining the impact of GAHT on ALT levels is not clearly elucidated. Further inquiry is imperative to clarify if there is an association.

### 4.4 Total bilirubin

Changes in total bilirubin (Tbili) have not been extensively studied in adults receiving GAHT (Table 5). In a small study of 11 adults on masculinizing HT, Tbili increased from 0.7 to 1.2 mg/dL (p = 0.01) in greater than 24 months (37). Another study revealed a reduction in Tbili in adults on 6 months of feminizing HT (0.5–0.4 mg/dL; *p* < 0.001) (35). At present, the existing body of research on the effects of GAHT on total bilirubin levels is far too scarce to form definitive conclusions.

### 4.5 Albumin

Minimal studies have explored shifts in albumin levels during the course of GAHT (Table 5). There does not appear to be any impact on albumin levels in adults on masculinizing hormone regimens (21, 35). In adults receiving feminizing HT, albumin has been shown to decrease slightly, however shifts were not clinically significant (21, 35). Further exploration of the impact of GAHT on albumin levels is warranted to address gaps in knowledge.

### 4.6 Conclusion

The hepatic system is complex with many important nutrition-relevant functions and thus it is important to gain insight on the impact of GAHT on liver function. While some of the existing research points to potential shifts in hepatic biochemical markers, there is not adequate evidence to support consistency in these findings. This topic warrants deeper exploration of large, homogenous samples to clarify the impact of GAHT on hepatic laboratory values.

## 5 Impact of GAHT on renal biochemical markers

Hyperkalemia is a known risk for estrogen-based regimens (8). It is recommended that individuals who are pursuing feminizing HT and are prescribed spironolactone as an anti-androgen have their electrolytes monitored every 3 months for the first year of HT with particular attention to serum potassium, BUN, and creatinine levels and then monitored every year (7, 9). When evaluating serum creatinine levels for individuals on GAHT, it is recommended that the male reference value is used as the upper limit of the normal reference range for those on masculinizing or feminizing hormone regimens. This is to account for the potential retention of muscle mass in transgender women and the increase in lean mass in transgender men (9).

### 5.1 Creatinine

Changes in serum creatinine (Cr) observed during GAHT have been well-documented in the literature (Table 6). In several studies, an increase in creatinine has been reported with the use of masculinizing HT (21, 25, 27, 29, 30, 35, 38) and this shift may manifest as early as 3 months following HT initiation (21, 25, 29, 30). There are also consistent results across several studies examining the impact of feminizing HT showing a reduction in serum Cr levels (21, 25, 27, 29, 30, 35, 38) which can also be observed at 3 months post-initiation (21, 25, 29, 30). Minimal evidence exists to support a change in serum Cr in adolescents and young adults on GAHT. In one study of this age group, no changes to Cr were observed (59), while in a later study, a rise in Cr was detected in those on a masculinizing hormone regimen and a reduction for those on a feminizing hormone regimen (64). Based on current research, an increase in creatinine may be anticipated with masculinizing HT and a reduction with feminizing HT.

**Table 6:** A summary of the changes in renal biochemical data in adults receiving masculinizing or feminizing hormone therapy.

References	Number of participants	Changes in biochemical values							
		Masculinizing HT				Feminizing HT			
		Cr	K	BUN	eGFR	Cr	K	BUN	eGFR
Chen et al. (21)	30 transmen; 29 transwomen	↑	–	–	–	↓	–	–	–
Fernandez and Tannock (25)	19 transmen; 33 transwomen	↑	–	–	–	↓	NC	–	–
Humble et al. (27)	150 transmen; 152 transwomen	↑	NC	NC	–	↓	↑	↑	–
Liu et al. (29)	65 transmen; 45 transwomen	↑	–	–	↓	↓	–	–	↑
Maheshwari et al. (30)	24 transmen; 84 transwomen	↑	–	–	–	↓	–	–	–
SoRelle et al. (35)	62 transmen; 87 transwomen	↑	NC	NC	–	↓	NC	NC	–
Wierckx et al. (38)	53 transmen; 53 transwomen	↑	–	–	–	↓	–	–	–

HT, hormone therapy; Cr, creatinine; K, potassium; BUN, blood urea nitrogen; eGFR, estimated glomerular filtration rate; NC, no significant change; ↑, significant increase; ↓, significant reduction.

### 5.2 Potassium

Serum potassium (K) levels are not well-studied in adults who receive GAHT (Table 6). From the available evidence, it does not appear that masculinizing HT has a significant effect on serum K (27, 35). A few studies have also documented no impact on serum K values with feminizing HT with spironolactone as the androgen antagonist (25, 35). In one study, a rise in serum K was observed at 6 months (3.9 vs. 4.1 mEq/L; *p* < 0.001) and 12 months (3.9 vs. 4.2 mEq/L; *p* < 0.001) following initiation of feminizing HT (27). Although these biochemical were statistically significant, they lack clinical significance. It is important to note that most of the subjects on feminizing HT in this study (27) were also concomitantly receiving spironolactone as an androgen antagonist. Spironolactone also functions as a potassium-sparing diuretic and was likely responsible for the potassium retention. A paucity of research exists exploring the impact of GAHT regimens on serum potassium levels. Further exploration on specifically the impact of spironolactone on the potassium levels of individuals undergoing feminizing HT may be essential to provide informed guidance for monitoring potassium levels.

### 5.3 Blood urea nitrogen

Blood urea nitrogen (BUN) levels in individuals undergoing GAHT have not been well-researched (Table 6). The minimal available evidence suggests no significant changes in BUN for those on a masculinizing hormone regimen (27, 35). There are mixed results from two studies evaluating BUN in adults on feminizing HT, with one study showing no impact (35) and the other with a slight increase in BUN (12 vs. 13 mg/dL; *p* < 0.05) at 6 months following HT initiation (27). Although this is a statistically significant rise, the change does not appear to be clinically significant. Based on the results of one study in which the BUN levels of adolescents and young adults on GAHT were assessed, there was no significant change detected (59). More research is necessary to adequately explore the influence of GAHT on BUN levels.

### 5.4 Estimated glomerular filtration rate

Estimated glomerular filtration rate (eGFR) has not been researched extensively among individuals prescribed GAHT (Table 6). Liu et al. (29) evaluated the impact of GAHT on eGFR and observed a progressive and significant reduction in eGFR in adults on a masculinizing hormone regimen at several timepoints during HT with an overall shift from 97.6 mL/min/1.73 m^2^ at baseline to 88.0 mL/min/1.73 m^2^ at 12–24 months following initiation of HT (*p* < 0.001). A slight increase in eGFR was also detected in subjects on a feminizing HT regimen for 6–12 months (115 vs. 118 mL/min/1.73 m^2^; *p* = 0.04), however this shift was minimal and not of clinical significance (29). Data on changes in eGFR levels during GAHT are extremely scarce and limited to one study in this review and therefore it is not feasible to draw any solid inferences. Additional studies focused on serial measures of eGFR during GAHT are warranted to gain a better understanding.

### 5.5 Conclusion

In alignment with the expected increase in muscle mass that is often observed in individuals pursuing masculinizing HT, a rise in serum creatinine is also expected. Due to the reduction of lean mass that often manifests with feminizing regimens, a subsequent reduction in creatinine is anticipated. Research on the influence of GAHT on potassium levels is inconclusive. Because of the pharmacological effects of spironolactone resulting in potassium reabsorption, expansion of research efforts on the impact of estrogen-based HT with spironolactone on serum potassium levels would provide more evidence-based guidance for monitoring this important electrolyte during feminizing HT. Inadequate evidence exists to support anticipated changes in BUN or eGFR, and further inquiry would address the gaps in understanding the relationship between GAHT and these important biochemical measures.

## 6 Discussion

Working with TGGD individuals receiving GAHT can pose challenges for nutrition professionals if they are unaware of the biochemical shifts that may be anticipated with HT. Based on the studies reviewed in which serum lipids were evaluated, it may be that some individuals will experience stable lipid levels, while others will exhibit unfavorable or favorable changes to their lipid panel, often observed with masculinizing or feminizing HT, respectively. Evidence available on biochemical data related to glycemic control and insulin resistance is emerging, however it is not adequate to draw solid conclusions. An increase in hemoglobin, hematocrit, and RBC count with masculinizing HT and a reduction with feminizing HT, on the other hand, is well-supported in the available literature and should be anticipated. Most research exploring the impact of GAHT on biochemical measures evaluating hepatic function supports that there may be no significant change observed. Although in some studies a rise in liver enzymes was reported for those on masculinizing hormone regimens and a reduction observed for individuals receiving feminizing HT. As for renal function, serum creatinine was consistently observed to increase with masculinizing HT, and to decrease with feminizing HT. It may also be important to closely monitor potassium levels if spironolactone is prescribed. Understanding the impact of GAHT on nutrition-relevant biochemical measures can assist nutrition professionals as they assess the nutritional needs of TGGD patients. Knowing the expected biochemical disturbances associated with GAHT can help rule out nutritional etiologies as well as provide an opportunity to offer proactive interventions. Future research may explore whether medical nutrition therapies can mitigate undesirable biochemical changes anticipated with GAHT. In addition, nutrition professionals may be unsure of which sex specific standards to use when interpreting assessment data. Anthropometric measures (body fat percentage, waist circumference, waist-to-hip ratio, body mass index-for-age percentiles) and certain biochemical data and medical tests (HDL-C, cholesterol to HDL-C ratio, hemoglobin, hematocrit, red blood cell count, ferritin, creatinine, alkaline phosphatase, bone mineral density) rely on sex-specific ranges for interpretation or diagnosis. Nutrition professionals may utilize values aligned with sex assigned at birth for patients who have not yet initiated HT, individualize their assessment based on the patient’s stage of medical transition, or apply a range (6). A limitation of this narrative review was that the researchers did not report in detail all of the approaches taken by the original authors of each study. Future research is needed to determine the precision of these strategies for patients at various stages of medical transitioning with GAHT.

The current body of literature exploring the impact of GAHT on nutrition-relevant biochemical measures is vastly heterogenous in the methodologies applied, including the prescribed hormone regimens and participant exclusion criteria. Much of the research is also retrospective with modest sample sizes. Larger prospective, multi-center studies should be conducted to further explore this emerging area of research and validate findings from earlier studies. This may facilitate a deeper comprehension of the expected alterations and the most efficacious strategies for their management.

## 7 Conclusion

Biochemical shifts related to cardiometabolic, hematologic, hepatic, and renal health are anticipated with masculinizing and feminizing HT. Nutrition professionals can be attuned to these expected shifts when caring for transgender patients on HT and can apply strategies for interpreting sex-specific biochemical data. Ongoing research is needed to inform optimal nutrition care for transgender patients on GAHT.

## Author contributions

JW: Conceptualization, Writing – original draft, Writing – review & editing. WL: Conceptualization, Writing – review & editing.
